# Identification and characterization of lipopolysaccharide binding protein (LBP) as an estrogen receptor α specific serum biomarker

**DOI:** 10.3109/1354750X.2012.654406

**Published:** 2012-02-03

**Authors:** Michael J Chisamore, Kwok-lam Karen Hong, Chun Cheng, Stephen E Alves, Susan P Rohrer, Hilary A Wilkinson

**Affiliations:** 1 Department of Molecular Endocrinology, Merck Research Laboratories Rahway, NJ, West Point, PA and Seattle, WA, USA; 2 Department of Molecular Profiling, Merck Research Laboratories Rahway, NJ, West Point, PA and Seattle, WA, USA

**Keywords:** Serum protein, steroid nuclear receptor, endocrine, estradiol

## Abstract

Estrogen Receptor a (ERα) and Estrogen Receptor β (ERβ) are steroid nuclear receptors that transduce estrogen signaling to control diverse physiological processes linked to reproduction, bone remodeling, behavior, immune response and endocrine-related diseases. In order to differentiate between ERα and ERβ mediated effects *in vivo*, ER subtype selective biomarkers are essential. We utilized ERα knockout (AERKO) and ERβ knockout (BERKO) mouse liver RNA and genome wide profiling to identify novel ERα selective serum biomarker candidates. Results from the gene array experiments were validated using real-time RT-PCR and subsequent ELISA's to demonstrate changes in serum proteins. Here we present data that Lipopolysacharide Binding Protein (LBP) is a novel liver-derived ERα selective biomarker that can be measured in serum.

## Introduction

Estrogen receptors (ERs) play a critical role in the development and maintenance of many tissues in the body. There are two known members of the estrogen receptor family, ERα and ERβ, encoded by distinct genes. ERα and ERβ DNA Binding Domain's (DBD) share 97% amino acid homology, while ligand binding domains (LBD) share 61% amino acid similarity. Both ligand-activated receptors bind to the same estrogen response element (ERE) and have comparable affinities for 17β-estradiol (E2). ERα and ERβ are steroid nuclear receptors which mediate effects of estrogen linked to reproduction, bone remodeling, behavior, immune response and endocrine-related diseases (Ariazi et al., 2006, Mueller and Korach, 2001).

The relative functions of ERα and ERβ have been investigated through the use of ERα, ERβ, and ERα/ERβ knockout mouse models. By this approach, null mutants of one or both of the ERs can be correlated to tissue specific functional changes of the distinct roles of ERα and ERβ (Dupont et al., 2000, Mueller and Korach, 2001). From these studies, it is apparent that ERα plays a critical role in reproductive physiology and bone remodeling while ERβ is important for ovarian cell differentiation and function. Further, high expression levels of ERβ in the CNS (Mitra et al., 2003) and data from knockouts indicate behavioral phenotypes (Rocha et al., 2005) suggesting an important role for ERβ in CNS function. Both ERα and ERβ have been implicated in modulating anti-inflammatory response in the periphery and CNS (Brown et al., 2010, Harris et al., 2003, Staples et al., 1999, Vegeto et al., 2003). While it has been established that knockout mice serve as a valuable tool to study the role of ERs *in vivo* (Couse and Korach, 1999), these models are not without shortcomings. For example, minor physiological responses may still take place as a result of the minute residual amounts of ERα mRNA transcripts in some AERKO mice (Couse et al., 1995). ER subtype specific selective ligands have also been utilized as tools to complement ER knockout mouse studies (Katzenellenbogen et al., 2000, Stauffer et al., 2000, Sun et al., 2002).

In order to confirm novel ERα or ERβ specific small molecule ligand activity, ER subtype selective serum biomarkers are essential research tools. In this study, a biomarker is defined as a protein that can be objectively measured by ELISA and used as an indicator of biologic response upon ER activation. In the present study, we have used liver RNA from mice lacking either ERα or ERβ, in microarray experiments to identify novel ERα selective serum biomarkers (Krege et al., 1998, Lubahn et al., 1993). Results from the gene array experiments were validated *in vivo* using real-time RT-PCR and subsequent ELISAs to demonstrate changes in serum biomarkers. We present data that LBP is a novel ERα selective biomarker that can be measured in serum. It was previously reported that estrogen can regulate rat liver LBP mRNA (Ikejima et al., 1998) and our current work demonstrate that this is an ERα driven event and that changes in LBP mRNA in the liver translate to changes in detectable protein in serum.

## Materials and methods

### 17-β-Estradiol and propyl-pyrazole-triol treatment of mice and rats

All animals were maintained in accordance with institutional Animal Care and Use guidelines. Genotype of the knockout mice was confirmed by PCR. Ovariectomized, 15 week old, c57BL (Taconic), Estrogen Receptor β (BERKO) (Krege et al., 1998), and Estrogen Receptor a (AERKO) (Lubahn et al., 1993), mice were treated (sc) daily with 0.2mg/kg 17-β-estradiol, 0.1, 1.0, and 10.0 mg/kg propyl-pyrazole-triol (Stauffer et al., 2000) or sesame oil vehicle for 4 or 14 days. Animals were euthanized, blood was drawn (via cardiac puncture), samples were incubated on ice for 15min, centrifuged at 12,000 g for 15 min, and serum was collected. The uterus was excised, trimmed of fat and connection tissue, and weighed. Additionally, ovariectomized 12 week old Sprague-Dawley CD rats (Charles River Laboratories, Wilmington, MA) were treated (sc) daily with 0.012, 0.05, and 0.2 mg/kg 17-β-estradiol or sesame oil vehicle for 3 days. Rats were euthanized and the uterus was collected and weighed as a bioassay for ERα activity. All 17-β-estradiol and propyl-pyrazole-triol mouse and rat studies are described in Supplementary Table SI.

### RNA extraction and cDNA synthesis

Total RNA from livers of mice and rats were isolated according to the manufacturer's instructions using Tri Reagent (Molecular Research Center, Cincinnati, OH). The RNA samples were treated with DNase I (Ambion, Grand Island, NY) and cDNA was synthesized using High-Capacity cDNA Archive Kit (Applied Biosystems, Foster City, CA).

### Quantitative real-time reverse transcription-PCR

Real-time RT-PCR was repeated three times in triplicate performed using the ABI 7900 HT Sequence Detection System (Applied Biosystems). The PCR conditions were 50°C for 2 min, 95°C for 10 min followed by 40 cycles of 95°C for 15 s and 60°C for 1 min. Primer/probe sets for mouse LBP (Mm00493139), rat LBP (Rn00567985_m1), and 18S rRNA endogenous control (4308329) were purchased from Applied Biosystems. The housekeeping gene 18S rRNA was used as the internal quantitative control for normalization. Relative gene expression was calculated with the ΔΔCt method as outlined in the Applied Biosystems User Guide.

### Statistics for uterine weight, real-time RT-PCR, and ELISAs

Error bars represent standard error of the mean (SEM) between replicates of a given experiment. Comparisons between two groups were made by analysis of variance (ANOVA) followed by a student *t*-test at 0.05 significance level with *p* values indicated.

### Gene expression profiling

Fluorescence-labeled cRNA transcribed from total RNA isolated from mouse liver (14 day E2 treatment experiment) were hybridized to DNA oligonucleotide microarrays as described previously (Hughes et al., 2001). Briefly, 5 μg of total RNA from an individual sample were used to synthesize dsDNA by RT. cRNA was produced by *in vitro* transcription and post-transcriptionally labeled with either Cy3 or Cy5. Reference and experimental cRNA samples were competitively hybridized to the Rosetta/Merck Mouse 25k v1.9 microarray which represents 22,700 genes (Tu et al., 2009). To minimize bias created by dye selection, for each comparison, two hybridizations were done with each cRNA sample pair using a fluorescent dye reversal strategy. After hybridization, arrays were scanned and fluorescence intensities for each probe were recorded. Ratios of transcript abundance in experimental versus control samples were calculated with normalized intensity data. Gene expression data analysis was done either with the Rosetta resolver gene expression analysis software (Version 5.1 Rosetta Biosoftware) or Matlab (Version 7, The Mathworks, Natick, MA). For each gene sequence on the arrays, statistical significance of differential gene expression was calculated according to the following equation: *p* value = 2 ×(1 − *Erf*(|*xdev*|)). Where Erf is the error function for a Gaussian distribution of zero mean and xdev is the adjusted difference in fluorescence intensities between Cy3 and Cy5 intensities as calculated by the equation: where *r* is the Cy5 intensity, *g* is Cy3 intensity, and σ is the error associated with the respective channel.

### Lipopolysaccharide binding protein expression measurement

LBP ELISA Test Kit (Cell Sciences, Norwood, MA) specific for mouse LBP was performed according to the manufacturer's protocol. ELISAs were repeated three times utilizing duplicate serum samples.

## Results

### E2 increased uterine weight in c57BL and BERKO but not AERKO mice

**Figure 1:**
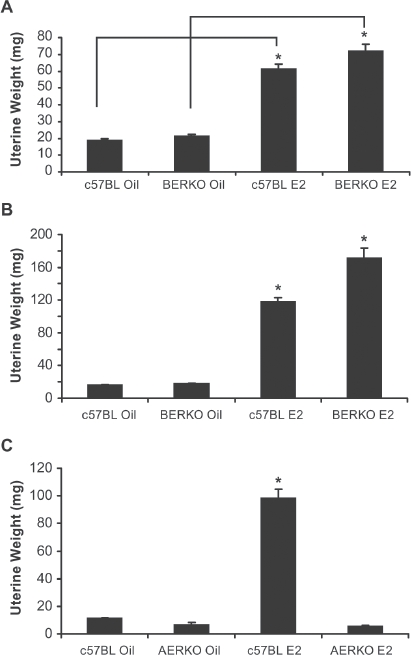
Four and fourteen day E2 dosing regimens lead to uterine weight increases in c57BL and BERKO but not AERKO mice. Uterine weight of c57BL and BERKO mice treated for (A) four days exhibited significant weight increases of 3.2 and 3.4 fold respectively. (B) Fourteen day treatment resulted in c57BL E2 treated mice resulted in a 7.3-fold significant increase while BERKO E2 treated mice had a 9.5-fold significant increase. (C) c57BL E2 treated mice for 14 days resulted in an 8.7-fold significant increase while AERKO E2 exhibited similar weights to AERKO vehicle treated mice. Error bars indicate standard error, *p < 0.001. Lines indicate pairwise comparisons.

Prior to performing microarray experiments, ERα knockout (AERKO) and ERβ knockout (BERKO) mouse models were validated by changes in uterine weight, a well-established bioassay of ERα-mediated proliferative effects (Owens and Ashby, 2002). C57BL (control) and BERKO mice were treated with vehicle or E2 for 4 (Figure 1A) or 14 days (Figure 1B). c57BL and BERKO mice treated with E2 at both time points had significant (*p* < 0.001) increases in uterine weight. At 4 days of treatment, c57BL E2 treated mice showed a 3.2-fold increase versus c57BL vehicle treated while BERKO E2 treated mice showed a similar increase, 3.4-fold when compared to BERKO mice treated with vehicle. Likewise, at 14 day treatment, c57BL E2 treated mice led to a 7.3-fold increase versus c57BL vehicle-treated mice while BERKO E2 treated mice had a 9.5-fold increase when compared to BERKO mice treated with vehicle. Additionally, C57BL (control) and AERKO mice were also treated with vehicle or E2 for 14 days (Figure 1C). WT mice treated with E2 exhibited an 8.7-fold significant (*p* < 0.001) increase versus vehicle treatment while AERKO mice did not respond to E2 treatment in uterine weight, as they exhibited uterine weights similar to WT mice treated with vehicle.

### Global gene expression microarray analysis in liver of C57BL, AERKO and BERKO mice afteri 4 day E2 treatment

To differentiate between ERα and ERβ effects *in vivo* and to identify novel ERα selective serum biomarker candidates, we profiled AERKO and BERKO mouse liver RNA in microarray experiments. C57BL, BERKO, and AERKO mice were treated with 0.2 mg/kg 17-β-estradiol or sesame oil vehicle for 14 days and ERα specific genes were identified on the basis of being differentially expressed in c57BL (control group) and BERKO but not in AERKO mice.

**Figure 2:**
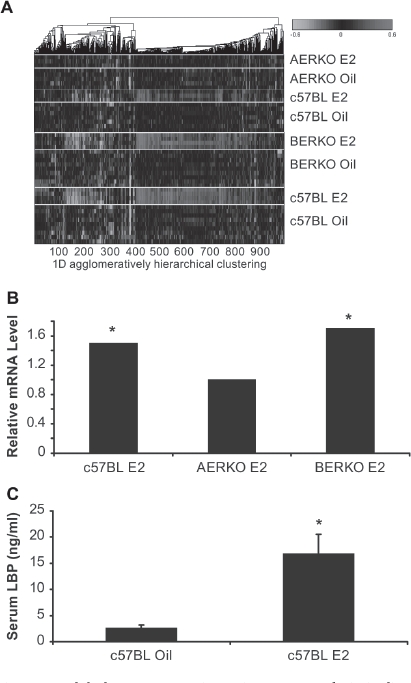
Global gene expression microarray analysis in liver of C57BL, AERKO and BERKO mice after 14 day E2 treatment identify potential ERα target genes. (A) Heat Map of changes in mRNA levels in liver of C57BL, AERKO and BERKO by microarray analysis after 14 day E2 treatment. ANOVA analysis was performed to identify unique patterns of gene regulation based on ER status followed by agglomerative clustering analysis (Matlab). Each row represents the different mouse models possessing different ER status and gene regulation. Up-regulated genes (pink), down-regulated genes (aqua), and genes showing no change verses control (black). (B) Fold change of LBP in mouse liver mRNA from c57BL and BERKO mice treated with E2 showed significant increases in LBP mRNA expression (1.5 and 1.7-fold respectively) while AERKO E2 did not change. (C) Change in serum LBP protein measured by ELISA (see materials and methods) when c57BL mice were treated with E2 for 14 days, a significant (*p* < 0.001) increase of 6.5-fold in serum LBP was demonstrated compared to the vehicle group. Error bars indicate standard error, *p < 0.001.

ANOVA analysis was performed to identify unique patterns of gene regulation based on ER status: c57BL (ERα/ERβ, +/+); AERKO (ERα/ERβ, -/+); BERKO (ERα/ ERβ, +/-) and 14 day E2 treatment. Agglomerative clustering analysis was performed using matlab (Figure 2A). Each row represents mouse models, ER status, and ER regulated genes. Up-regulated genes are depicted in pink, down-regulated genes are aqua, and genes showing no change verses control are black. Heat map changes demonstrate unique gene signatures illustrating patterns of potential ERα target genes in the liver. Examination of individual genes of interest (serum proteins) identified Lipopolysacharide Binding Protein (LBP). c57BL and BERKO mice treated with E2 showed significant (*p* < 0.001) increases in LBP mRNA expression (1.5 and 1.7-fold, respectively) while expression was not regulated by E2 in AERKO mice. (Figure 2B). WT mice treated with E2 for 14 days, exhibited a significant (*p* < 0.001) increase (6.5-fold) in serum LBP compared to the vehicle treated group (Figure 2C).

### C57BL mice treated with ERα agonist propyl-pyrazole-triol (PPT) exhibit increased LBP expression

**Figure 3:**
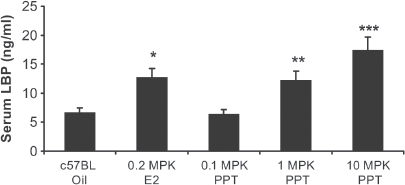
Dose-dependent increase in serum LBP with an ERα agonist: propyl-pyrazole-triol (PPT). c57BL mice treated with O.lmg/kg PPT did not result in a significant difference in LBP serum levels (when compared to vehicle, measured by ELISA), l.Omg/kg PPT led to a 1.9-fold and lOmg/kg PPT demonstrated a 2.6-fold significant increase in serum LBP. A significant 1.9-fold increase of serum LBP was also demonstrated when the mice were treated with 0.2 mg/kg E2. Error bars indicate standard error of the mean. *, *p* = 0.002; **, *p* = 0.003; and ***, *p* < 0.001.

Upon demonstrating E2 treatment leads to increased LBP mRNA and protein expression (Figure 2B and 2C), c57BL mice were dosed for 4 days with PPT (propyl-pyrazole-triol), a highly selective ERα ligand that binds to ERα with a 400-fold affinity higher then ERβ (Stauffer et al., 2000). Dose dependent significant increases in serum LBP protein were demonstrated when mice were treated with PPT. While 0.1 mg/kg PPT treatment did not lead to a difference in LBP serum levels (when compared to vehicle), 1.0 mg/kg PPT led to a 1.9-fold (*p* = 0.003) and lOmg/kg PPT led to a 2.6-fold (*p* < 0.001) increase in serum LBP. Additionally, a significant (*p* = 0.002) 1.9-fold increase of serum LBP was demonstrated when the mice were treated with 0.2 mg/ kg E2 (Figure 3).

### Expression of functional ERα but not ERβ is required for up regulation of LBP by E2 or PPT treatment

**Figure 4:**
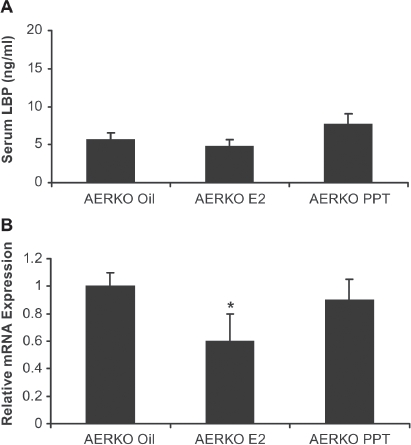
Four day 17β-estradiol and PPT treatments have no effect on serum LBP in AERKO mice while E2 lowers LBP mRNA in AERKO mice livers. (A) AERKO mice treated with E2 or PPT for 4 days result in no difference in serum LBP levels when compared to vehicle (measured by ELISA). B) E2 treatment resulted in a 40% significant decrease of LBP mRNA (measured by real-time RT-PCR, see materials and methods) in AERKO mice liver while the ERα subtype specific agonist, PPT did not demonstrate a significant effect (compared to vehicle). Error bars indicate standard error of the mean. *, *p* = 0.01.

**Figure 5:**
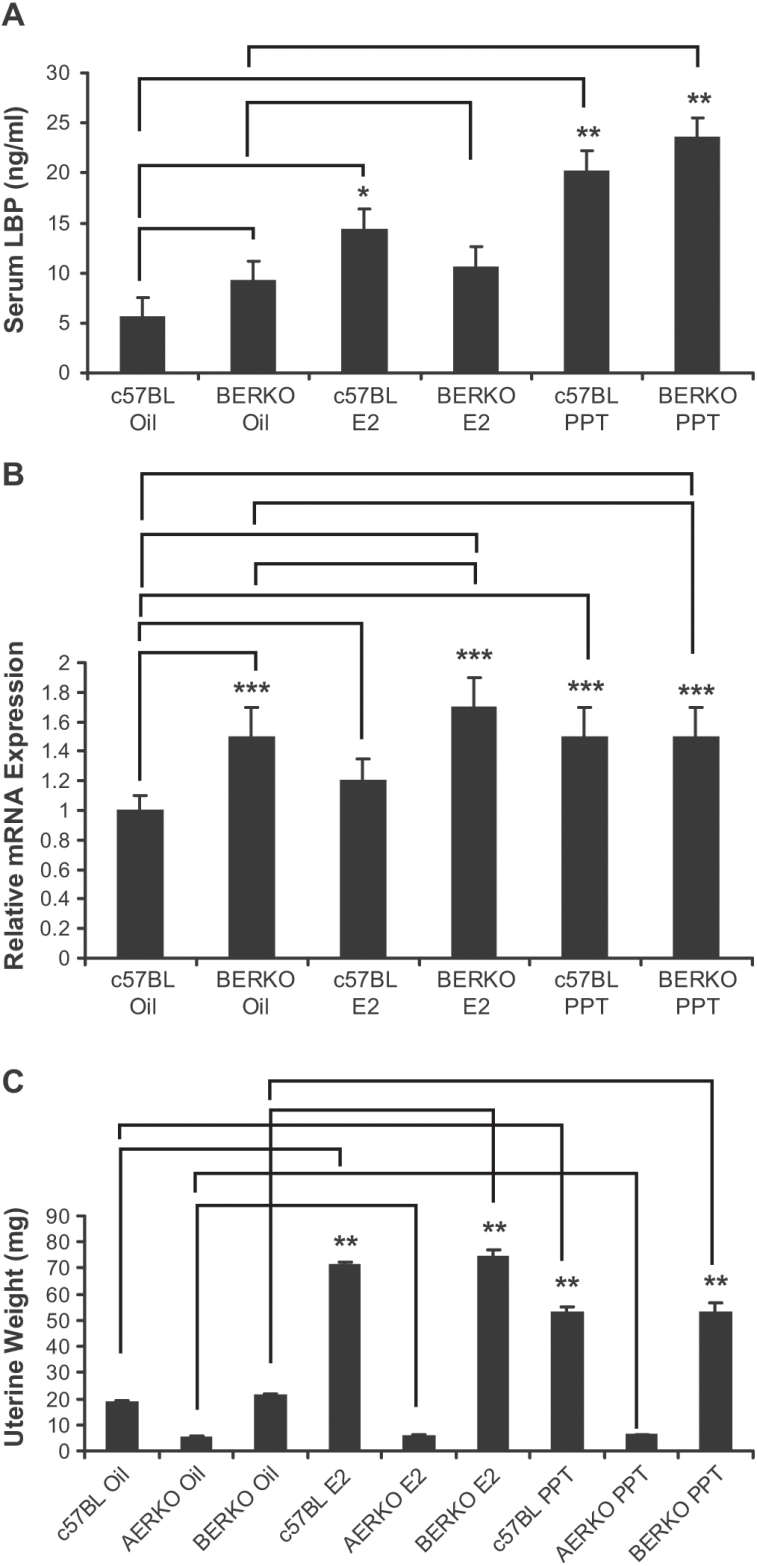
BERKO mice treated with PPT for 4 days increase serum LBP, liver mRNA LBP expression, and uterine weight. (A) c57BL and BERKO mice treated with vehicle, E2, and PPT for 4 days. c57BL mice treated with E2 led to a significant increase of 2.6-fold in serum LBP (measured by ELISA) when compared to the vehicle (oil) group. c57BL mice treated with PPT demonstrated asignifi cant increase of 3.6-fold in serum LBP compared to the vehicle (oil) group. BERKO mice treated with E2 (10.6ng/mL) resulted in a slight increase of serum LBP when compared to the BERKO's treated with the oil vehicle (9.2 ng/mL). BERKO mice treated with PPT showed a significant 2.6-fold increase in comparison to the BERKO vehicle (oil). (B) Mouse liver mRNA LBP expression was measured (real-time RT-PCR), a 1.2-fold increase was exhibited in c57BL mice treated with E2 when compared to the c57BL vehicle (oil) while a significant 1.5-fold increase was seen in c57BL mice treated with PPT when compared to the c57BL vehicle (oil) mice. BERKO mice treated with sesame oil demonstrated a significant 1.5-fold increase of LBP mRNA expression when compared to the c57BL mice treated with vehicle (oil). BERKO mice treated with E2 led to a 12% increase in LBP mRNA expression when compared to BERKO mice treated with vehicle (oil). BERKO mice treated with E2 compared to the c57BL mice treated with vehicle (oil) yielded a 1.7-fold significant increase. BERKO mice treated with PPT and BERKO mice treated with vehicle (oil) express approximately the same amount of LBP mRNA. BERKO mice treated with PPT express a 1.5-fold significant increase compared to the c57BL treated vehicle (oil) mice. (C) c57BL mice treated with E2 resulted in a 3.8-fold significant increase in uterine weight while PPT treatment resulted in a 2.8-fold significant increase (compared to c57BL mice treated with sesame oil vehicle). BERKO mice treated with E2 or PPT demonstrated 3.5 and 2.5-fold significant increases respectively (compared to BERKO mice treated with oil vehicle). AERKO mice treated with vehicle (oil), E2, or PPT led to no differences in uterine weight. Error bars indicate standard error of the mean. *, *p-* 0.004;**, *p* < 0.001; and***, *p* <0.05. Lines indicate pairwise comparisons.

In contrast to C57BL6 mice, serum LBP did not change with any treatment in AERKO mice (Figure 4A). E2 treatment significantly decreased (∼40%, *p* = 0.01) LBP mRNA in AERKO liver while the ERα selective agonist, PPT did not impact LBP expression (Figure 4B), thus suggesting an ER-β mediated effect on LBP lowering serum LBP was increased by both E2 (2.6 fold, *p* = 0.004) and PPT (3.6 fold, *p* < 0.001, Figure 5A) in C57BL6 mice. Interestingly, vehicle-treated BERKO mice exhibited a trend towards higher serum LBP levels (∼1.6 fold) compared to c57BL mice (Figure 5A). While a small, non-significant increase in serum LBP was observed in BERKO mice following E2 treatment (10.6 ng/mL), BERKO mice treated with PPT exhibited a significant (*p* < 0.001, 2.6-fold) increase in LBP (Figure 5A). Furthermore, E2 or PPT induced a small increase in liver LBP and mRNA expression (1.2-fold, 1.5 fold respectively) in c57BL mice (*p* < 0.05) (Figure 5B). BERKO mice treated with E2 showed a trend towards an increase (12%) in LBP mRNA expression. Similar to the vehicle-treated groups, BERKO mice treated with E2 (compared to c57BL mice treated with vehicle) yielded a 1.7-fold significant (*p* < 0.05) increase (Figure 5B). BERKO mice treated with PPT when compared to the BERKO mice treated with vehicle expressed approximately the same amount of LBP mRNA, but when the BERKO mice treated with PPT were compared to the c57BL treated vehicle mice, a significant (*p* < 0.05) 1.5-fold increase in LBP mRNA expression is exhibited (Figure 5B). Taken to together these data represent that LBP expression is an ERα driven effect, BERKO mice exhibit a higher baseline expression level of LBP, and ERβ absence leads to higher LBP expression. E2 or PPT treatment increased uterine weight significantly in c57BL mice (3.8 and 2.8-fold respectively) (*p* < 0.001). BERKO mice exhibited significant increases (3.5 and 2.5-fold respectively) (*p* < 0.001). However, AERKO mice did not respond to E2 or PPT treatment on uterine weight (Figure 5C), confirming uterine proliferation as an ERα driven response.

### 17β-estradiol increases LBP mRNA expression in rat liver

**Figure 6:**
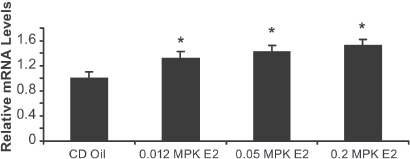
17β-estradiol increases LBP mRNA in rat liver. Sprague-Dawley CD rats were treated vehicle (oil), 0.012, 0.05, and 0.2 mg/ kg E2 for 3 days. mRNA LBP expression measured (by real-time RT-PCR) in the liver yielded a dose dependent significant increase of 1.3,1.4, and 1.5-fold (compared to oil vehicle) when dosed with, 0.012, 0.05, and 0.2mg/kg E2 respectively. Error bars indicate standard error of the mean. *, *p* < 0.05.

To confirm increase of LBP mRNA expression by E2 was not mouse specific, we also studied LBP expression in Sprague-Dawley CD rats treated with vehicle, 0.012, 0.05, and 0.2 mg/kg E2 for 3 days. mRNA LBP expression measured in the liver exhibited a dose dependent significant (*p* <0.05) increase of 1.3, 1.4, and 1.5-fold respectively (Figure 6).

## Discussion

The mechanisms via which estrogens mediate their vast effects continue to be elucidated. It is well established that ERs mediate functional effects of estrogen that are associated with reproduction, bone remodeling, behavior, immune response and endocrine-related diseases. As medicine becomes more personalized, serum biomarkers of therapeutic drug targets (such as ERs), will become even more important.

LBP is a glycosylated 60 kDa serum protein that is primarily produced in the liver (Ramadori et al., 1990). LBP plays a central role in the response to LPS by accelerating the binding of lipopolysacharide (LPS) monomers and inducing cytokine production through a CD14 dependent pathway (Vollmer et al., 2009, Wright et al., 1990, Wurfel et al., 1997). LBP acts as a lipid transfer protein by catalyzing the transfer of LPS monomers from aggregates to CD14 (Hailman et al., 1994, Tobias et al., 1995). LBP has a high affinity for LPS and other membrane proteins (Tobias and Ulevitch, 1993) and it is constitutively released into the bloodstream upon type 1 acute phase stimulation (Schumann and Latz, 2000). LBP co-purifies with HDL particles (Wurfel et al., 1994) and packages LPS into complexes of high density lipoprotein (HDL) (Wurfel et al., 1995) where LPS is neutralized thereby protecting against LPS toxicity (Schumann and Latz, 2000, Skarnes, 1966). LBP has been reported to be a biomarker for the diagnosis of local bacterial infection (Vollmer et al., 2009) and LBP is involved in LPS-induced tight junction disruption and increased permeability in bile duct epithelial cells (Sheth et al., 2007).

ERs have been reported to alter both adaptive and innate immune responses and gender-based differences in infectious disease susceptibility are thought to result in part from robust effects of estrogen on proin-flammatory and anti-inflammatory cytokine expression (Cunningham and Gilkeson, 2011). Both ERα and ERβ have been reported to suppress proinflammatory cytokine expression (Cvoro et al., 2008). Rettew et al have shown ovariectomy of mice decreases circulating LBP levels (Rettew et al., 2009). In addition, they showed alterations in cell surface expression of a key effector of immune response to microbial infection (e.g. TLR4) in sentinel immune cells. Interestingly, treatment of ovariectomized animals with 17-β-estradiol increased circulating LBP levels and this corresponded to an increase in endotoxin susceptibility. Ikejima et al. in 1998 reported an increase in liver LBP mRNA as well as functionally active LBP in serum when female Sprague–Dawley rats were treated with 20 mg/kg estriol (Ikejima et al., 1998). These studies did not investigate the differential role of ERα and ERβ in regulating LBP expression and our work demonstrates the role of ERα in mediating the increase in circulating LBP. In this study, we have used molecular profiling of liver gene expression in mice with different ER status: c57BL (ERα/ERβ, +/+); AERKO (ERα/ERβ, −/+); BERKO (ERα/ERβ, +/−) to identify LBP as an ERα specific serum marker. We have verified changes in LBP gene expression by utilizing real-time RT-PCR and demonstrated changes in protein level by performing ELISA's. Additionally, uterine weight was utilized to monitor estrogen-mediated proliferative effects on the uterus. Along with LBP, Sex Hormone-Binding Globulin (SHBG), Monocyte Chemoattractant Protein-1 (MCP-1), Insulin-Like Growth Factor-Binding Protein-1 (IGFBP-1), Cholesterol 7-α-Hydroxylase (CYP7a1), Peroxisome Proliferator-Activated Receptor α (PPARα), and Small Heterodimer Partner (SHP) were identified from the microarray analysis as potential biomarkers. Candidates were required to be found in the blood and an ELISA needed to be available for study. Upon analysis of candidates, LBP was found to be the most robust and consistent serum biomarker.

In the liver, LBP mRNA is increased in response to E2 and this change is detected at both 4 days and 2 weeks of treatment. Serum levels of LBP protein are altered in the mouse in response to E2 and we detect up to a 6.5-fold increase in LBP serum protein within 2 weeks. We have demonstrated that the response to 17β-estradiol is mediated via ERα because LBP is altered in response to treatment with PPT, an ERα selective agonist in a dose-dependent manner. In addition LBP protein levels are not changed in AERKO animals in response to either E2 or the ERα selective agonist. It is likely treatment with an ERα selective agonist may result in hypersensitization to microbial infection which can be both harmful in supporting a robust susceptibility to infection by certain bacteria or viruses, however it has also been shown to be beneficial in providing strong protection against other types of bacterial infection (Rettew et al., 2009).

LBP levels in BERKO animals are altered in response to the ERα selective agonist but do not have significant responses to E2. This appears to be due to unusually high levels of LBP in untreated BERKO mice serum which may indicate that ERα is “more” active in the absence of ERβ; it is also possible that ERβ has a direct role in maintaining basal circulating levels of LBP. Our data suggests that LBP expression is an ERα driven effect. In the future, it will be important to study why knocking out ERβ (BERKO mice) led to increased LBP levels. Possible mechanisms of action include, with ERβ not present, there is more ERα present to drive LBP expression or ERβ may have repressive effects on ERα that usually keep LBP levels lower when ERβ is present. The respective roles of ERα and ERβ in mediating effects of estrogen on the innate immune system have been studied both with selective ligands and in knockout animal models (Cristofaro et al., 2006, Curran et al., 2001, Cvoro et al., 2008). These studies suggest a therapeutic effect of ERβ ligands in the treatment of sepsis. It is possible one advantage of ERβ selective ligands is they would not increase circulating LBP levels and/or may even lower endogenous levels.

Our approach of using an ERα selective agonist serves as a valuable complement to the use of ER knockout mice to explicate novel ER subtype specific biomarkers. We appreciate that there are limitations in utilization of the knockout model, in that the mice are genetically lacking either ERα or ERβ from early embryonic development, and thus, there is the potential for complicated developmental compensation networks at play that have not been completely elucidated (Carpenter and Korach, 2006, Hewitt et al., 2005, Pearce and Jordan, 2004) or could be comparatively studied in liver specific ERα or ERβ knockout models. Removal of only one of the ER subtypes abolishes the modulation of responses that may take place due to synergistic or antagonistic interactions between the two proteins that might also modify either an increase or decrease of expression due to ERα and ERβ crosstalk. Therefore, the use of an ERα subtype specific agonist provides an additional tool to aid in identifying subtype specific biomarkers.

In summary, our studies include independent verification by real-time RT-PCR and ELISA that our microarray results obtained have physiological and functional significance. We show that subtle liver LBP mRNA changes can lead to greater LBP protein expression changes detectable in serum. Lastly, our data suggest that LBP may have utility as an ERα selective serum biomarker in the clinic. This is very valuable given the importance of ERα and ERβ as therapeutic targets. LBP appears to be an ERα selective serum biomarker that can be utilized to study and confirm ER subtype specific ligands that elicit unique biological responses that could play a valuable role in developing novel endocrine therapies targeted to the estrogen receptor.
